# Household Transmission of Carbapenemase-producing *Klebsiella pneumoniae*

**DOI:** 10.3201/eid1405.071340

**Published:** 2008-05

**Authors:** Tamar Gottesman, Orly Agmon, Orna Shwartz, Michael Dan

**Affiliations:** * Edith Wolfson Hospital, Holon, Israel

**Keywords:** Carbapenemase, nosocomial infection, household transmission, Klebsiella pneumoniae, letter

**To the Editor:** Since its first description in 2001, carbapenemase-producing *Klebsiella pneumoniae* has become a frequent nosocomial pathogen in the eastern United States (*1*). This bacterium was introduced into Israel in 2005 and is endemic now in several hospitals in the country (*2*). We recently documented transmission of this organism within a household, the source being a debilitated patient who returned home after a long hospitalization.

A 73-year-old man had a urologic procedure (transurethral resection of the bladder neck) in a community hospital in early October 2007. He was initially evaluated on September 23, 2007, at an outpatient clinic where a routine urine sample was obtained for culture. Carbapenemase-producing *K*. *pneumoniae* was cultured. Identification and susceptibility testing of the isolate were completed by using the VITEK 2 system (bioMérieux, Marcy l’Etoile, France). *K*. *pneumoniae* carbapenemase was confirmed by using the modified Hodge test (*3*). Two repeat urine cultures grew the same organism; however, a stool culture was negative for carbapenemase-producing *K*. *pneumoniae*.

The medical history of the patient included hypertension and carcinoma of the prostate gland that was treated with high-intensity focused ultrasound in May 2007, followed by transurethral resection of prostate in June 2007. The 2 procedures were performed in 2 different private hospitals, and each required a 24-hour hospitalization. No carbapenemase-producing *K*. *pneumoniae* was documented in these hospitals. Two months before detection of carbapenemase-producing *K*. *pneumoniae*, the patient received a 1-week course of oral amoxicillin-clavulanate for presumed urinary tract infection, although urine culture obtained on July 29, 2007 was sterile. A repeat urine culture 2 weeks later (August 13, 2007) remained sterile.

Because the circumstances of strain acquisition and patient characteristics were not typical for epidemiology of carbapenemase-producing *K*. *pneumoniae* (*3*), he was further questioned about possible contacts of relevance. The patient disclosed that his wife, who had amyotrophic lateral sclerosis that required mechanical ventilation, had been hospitalized in a tertiary hospital in the Tel Aviv area for 9 weeks until July 19, 2007. After discharge, she has been staying at home where she was cared for by her son, sister, and nurses; the patient stated that he had limited contact with his wife (he did not participate in her care). The infection control unit of the tertiary hospital was contacted, and the name of the wife was identified in the hospital registry. Carbapenemase-producing *K*. *pneumoniae* was isolated from her urine on June 8, 2007.

Despite limited contact, the patient probably acquired carbapenemase-producing *K*. *pneumoniae* from his wife, who was a documented carrier of this organism. Because his early urine cultures (taken after his wife was discharged from hospital) were sterile, we can assume that the transmission of the organism occurred at their home. We cannot rule out that the strain was transferred by an intermediary, such as the couple’s son. It is unlikely that the organism was acquired at the private hospitals from which no case of carbapenemase-producing *K*. *pneumoniae* was reported (in Israel reporting carbapenemase-producing *K*. *pneumoniae* isolates to health authorities is mandatory). Also, the patient had 2 negative urine cultures.

Carbapenemase-producing *K*. *pneumoniae* is a recent addition to the pool of multidrug-resistant nosocomial pathogens. Most publications on this organism have focused on issues of structural and molecular epidemiology. Little is known regarding clinical characteristics and importance of infection with this organism. Until now, the strain has been recovered only from hospitalized patients with a longer hospital stay, those given multiple antimicrobial drug courses, and those mechanically ventilated (*3**,**4*). The strain can colonize the urinary, intestinal, and respiratory tracts, as well as wounds; bloodstream infection is associated with higher death rates than infection at other sites (*4*). Hand carriage is probably the biggest factor in transmission of extended-spectrum β-lactamase producers, and there is little evidence to suggest that carriers of carbapenemase-producing *K*. *pneumoniae* would be different. Environmental contamination plays a limited role in transmission of the organism (*3*). Caregivers should be aware that multidrug-resistant organisms of nosocomial origin can be transmitted in the community (*5*). Acquisition of such strains is probably of negligible importance in an otherwise healthy person. However, consequences may be different if the recipient of the strain is a debilitated patient.
